# Membrane Homeoviscous Adaptation in *Sinorhizobium* Submitted to a Stressful Thermal Cycle Contributes to the Maintenance of the Symbiotic Plant–Bacteria Interaction

**DOI:** 10.3389/fmicb.2021.652477

**Published:** 2021-12-17

**Authors:** Natalia Soledad Paulucci, Adriana Belén Cesari, María Alicia Biasutti, Marta Susana Dardanelli, María Angélica Perillo

**Affiliations:** ^1^Facultad de Ciencias Exactas, Físico-Químicas y Naturales, Departamento de Biología Molecular, Universidad Nacional de Río Cuarto, Río Cuarto, Argentina; ^2^Instituto de Biotecnología Ambiental y Salud (INBIAS), Consejo Nacional de Investigaciones Científicas y Técnicas (CONICET), Río Cuarto, Argentina; ^3^Facultad de Ciencias Exactas, Físico-Químicas y Naturales, Departamento de Química, Universidad Nacional de Río Cuarto, Río Cuarto, Argentina; ^4^Instituto para el Desarrollo Agroindustrial y de la Salud (IDAS), Consejo Nacional de Investigaciones Científicas y Técnicas (CONICET), Río Cuarto, Argentina; ^5^Facultad de Ciencias Exactas, Físicas y Naturales, Instituto de Ciencia y Tecnología de Alimentos (ICTA), Departamento de Química, Cátedra de Química Biológica, Universidad Nacional de Córdoba, Córdoba, Argentina; ^6^Instituto de Investigaciones Biológicas y Tecnológicas (IIBYT), Consejo Nacional de Investigaciones Científicas y Técnicas (CONICET), Córdoba, Argentina

**Keywords:** *Sinorhizobium meliloti*, homeoviscous adaptation, temperature change, plant-bacteria interaction, outer and inner membrane

## Abstract

Here, we estimate fast changes in the fluidity of *Sinorhizobium meliloti* membranes submitted to cyclic temperature changes (10°C–40°C–10°C) by monitoring the fluorescence polarization (*P*) of DPH and TMA-DPH of the whole cell (WC) as well as in its outer (OM) and inner (IM) membranes. Additionally, the long-term response to thermal changes is demonstrated through the dynamics of the phospholipid and fatty acid composition in each membrane. This allowed membrane homeoviscous adaptation by the return to optimal fluidity levels as measured by the PDPH/TMA-DPH in WC, OM, IM, and multilamellar vesicles of lipids extracted from OM and IM. Due to probe-partitioning preferences and membranes’ compositional characteristics, DPH and TMA-DPH exhibit different behaviors in IM and OM. The rapid effect of cyclic temperature changes on the *P* was the opposite in both membranes with the IM being the one that exhibited the thermal behavior expected for lipid bilayers. Interestingly, only after the incubation at 40°C, cells were unable to recover the membrane preheating *P* levels when cooled up to 10°C. Solely in this condition, the formation of threads and nodular structures in *Medicago sativa* infected with *S. meliloti* were delayed, indicating that the symbiotic interaction was partially altered but not halted.

## Introduction

*Sinorhizobium meliloti* are Gram-negative bacteria usually applied in the formulation of commercial inoculants because of their ability to promote the growth and yield of legume crops (e.g., *Medicago sativa*, alfalfa) through a symbiotic atmospheric nitrogen (N)-fixing relationship.

N is fundamental for the development of plants because it is needed for the synthesis of proteins and nucleic acids. To counteract the deficit of N in certain soils, alfalfa establishes N-fixing symbiosis with the bacterium *S. meliloti* through a series of continuous signal exchanges that results in structural changes of *Medicago* roots (Jones et al., 2007). The first structural change is the formation of tightly curled root hairs entrapping *S. meliloti* cells (Gage, 2004). The presence of bacterial cells inside curled root hairs leads to the formation of tube-like infection threads (Gage and Margolin, 2000) which extend toward the bases of root hairs, branch into multiple infection threads, pass layers of root cortical cells, and reach the developing nodule primordia (Kijne, 1992; Timmers et al., 1999). Nodule primordia continue to generate new plant cells and form elongated indeterminate alfalfa nodules (van Brussel et al., 2002). *S. meliloti* cells are released from infection threads into the cytoplasm of new nodule cells surrounded by a plant cell membrane in the small nodule invasion zone (Hirsch et al., 1983). Thus, if all internal conditions are favorable, *S. meliloti* cells differentiate into bacteroids and convert atmospheric N_2_ into NH_4_^+^.

Inoculation is the technology developed with the purpose of incorporating highly infectious and highly efficient rhizobia in legumes of agricultural interest. The inoculants must overcome two major problems inherent to living microorganisms: (1) loss of viability during short storage in the grower’s warehouse, and (2) long shelf life and stability of the product over a range of −5 to 30°C within the grower’s storage conditions.

During storage of the inoculants, bacteria experience fluctuations in temperature that are both regular (diurnal and seasonal) and random (as a result of physical disturbances of the environment). Most studies that address the issue of adverse environmental factors are designed so that different bacterial samples are exposed to either optimal or adverse conditions. However, there are few or no studies addressing different conditions on the same bacterial population to know the dynamics of the adaptation processes to environmental changes that bacteria can undergo in natural conditions.

Among environmental factors, temperature is the one exerting the strongest impact on the cell (WC) envelope of rhizobacterias (Paulucci et al., 2011, 2015; Cesari et al., 2016, 2018). Poikilothermic organisms, including bacteria do not control their temperature and must adapt their membrane lipid composition to maintain an optimal membrane fluidity level (Ernst et al., 2016). This adaptive response is termed “homeoviscous adaptation” (Sinensky, 1974) and in Gram-negative bacteria has been frequently studied on the WC envelope with a specific focus on the inner membrane (IM) (cytoplasmatic membrane). To our knowledge, except for a few studies on *Escherichia coli* and *Yersinia pseudotuberculosis* (Lugtenberg and Peters, 1976; Davydova et al., 2016), the role of the outer membrane (OM) in the thermal adaptation process has not received much attention, whereas in the case of rhizobacteria, this kind of data are null. In this work, we describe for the first time the biophysical behavior and mechanisms of homeoviscous adaptation due to cyclic temperature changes to maintain the OM and IM fluidity of a rhizobacteria.

The fluorescent probes 1,6-diphenyl-1,3,5-hexatriene (DPH) and its trimethylammonium derivative (TMA-DPH) are widely used to study the physical state of biological membranes (Konopasek et al., 2000; Balogh et al., 2011; do Canto et al., 2016). In typical lipid bilayers, DPH is known to penetrate in the membrane interior while its polar derivative, TMA-DPH, remains at the membrane surface within the lipid polar headgroup region (Lentz, 1989). However, when DPH is used as a probe in Gram-negative bacteria, the complex cell envelope may produce misleading results from fluorescence polarization experiments obtained in WC and OM, leading to erroneous interpretations. This is because the OM is a lipid bilayer containing phospholipids (PL), which are confined to the inner monolayer of the lipid bilayer, whereas the outer monolayer is mainly composed of lipopolysaccharides (LPS) (Bos et al., 2007). The hydrophilic polymeric moiety of LPS faces the outermost part of the cell and affects the partitioning of DPH in the core of the bilayer. DPH works well as a sensor of the behavior of the IM. In turn, the interpretation of the fluorescence polarization values of both probes in OM may be hindered by other physical phenomena such as solvent polarization and the molecular dynamics of the hydrophilic environment provided by the polysaccharide chains of LPS. There, TMA-DPH achieves a concentration higher than that of DPH and the highest with respect to other membrane regions.

Lipid components of the bacterial cell envelope largely influence the bacteria–plant symbiotic interaction process (Siebers et al., 2016). Phosphatidylcholine (PC) is a PL present in the membranes of bacteria of the rhizobiaceae family (Sohlenkamp et al., 2003; Basconcillo et al., 2009; Paulucci et al., 2011, 2013, 2014, 2015) and is essential for symbiotic interactions of rhizobia with legumes. For example, root nodule formation on soybean with efficient N fixation after infection with *Bradyrhizobium japonicum* depends on the presence of PC in the bacterium (Hacker et al., 2008). Although the process of symbiotic interaction between *S. meliloti* and alfalfa is deeply studied (Sieberer et al., 2005; Peck et al., 2006; Jones et al., 2007), to our knowledge, there are no studies that analyze the effects of environmental conditions exerted on biochemical changes in the cell envelope and the subsequent events of early colonization of alfalfa root simultaneously in the same samples.

In this work, we perform steady-state measurements of the DPH and TMA-DPH fluorescence polarization and fluorescence calculations to study the short-term effects of cyclic temperature changes (10°C–40°C–10°C) on the molecular dynamics of each membrane of *S. meliloti*, using WCs, spheroplasts, and isolated OM. Then, through lipid composition analysis, we study the homeoviscous response achieved by the bacteria in each membrane after the temperature change to establish if it could reverse the initial effects. In addition, we analyze the effects of cyclic temperature changes on early interaction events of *S. meliloti* with roots of *M. sativa*. Although homeoviscous adaptation was successfully achieved through changes in the lipid composition of OM and IM, the plant–bacteria interaction was affected after exposure of *S. meliloti* to 40°C.

Two main issues, the study of successive thermal changes within the same bacterial population and the dissection of OM and IM, differentiate our work from others appearing in the literature in the research field of thermal adaptation in bacteria membranes. Moreover, studying the thermal-induced changes in lipid composition of the *S. meliloti* envelope not only sheds light on its biochemical mechanisms of adaptation, but might also contribute to explain the thermal-mediated *Sinorhizobium* and *Medicago* crosstalk at the molecular level.

## Materials and Methods

### Materials

Thin layer chromatography (TLC) plates (silica gel GHLF, UV254, 250 micron) were purchased from Analtech, Inc. (Newark, DE, United States). Lipid standards were purchased from Sigma-Aldrich Co. (St. Louis, MO, United States). Fluorescent probes were from Invitrogen™, Thermo Fisher Scientific, Argentina. All reagents were of analytical grade and solvents of HPLC grade.

### Bacteria and Growth Conditions

*Sinorhizobium meliloti* 1021 was cultured at 28°C in Luria-Bertani (LB) medium until the stationary phase, then an aliquot of the culture was separated and the rest placed at 10°C for 24 h. Then, an aliquot was separated and the rest subjected to 40°C for 24 h. In the same way afterward, an aliquot was separated, and the rest was exposed for 24 h at 10°C again to complete the cooling–heating–cooling cycle (10°C–40°C–10°C). The aliquots of culture separated at each point of the cycle were centrifuged and the biomasses used for the different determinations.

### Isolation of Outer Membrane and Inner Membrane

Fractions of internal and external membrane were obtained according to the technique of Mizuno and Kageyama (1978). Cells were harvested by centrifugation at 8,000 rpm for 10 min at room temperature and washed with saline buffer pH 7. Cells (ca. 1.5 g wet weight) were suspended in 18 mL of ice cold 20% (w/v) sucrose. Ice cold reagents were slowly added to the suspension in an ice bath in the following order: 9 mL of 2 M sucrose, 10 mL of 0.1 M Tris–HCl (pH 7.8), 0.8 mL of 1% Na-EDTA (pH 7.0), and 1.8 mL of 0.5% lysozyme. Then, the mixture was warmed to 30°C within a few minutes and kept at that temperature for 60 min. During the incubation, the mixture became viscous due to the cell disruption. Then, the suspension was centrifuged at 13,000 rpm for 15 min at 30°C to obtain the spheroplasts in the pellet. Crude OM were recovered from the supernatant by centrifugation at 30,000 rpm for 60 min. The spheroplasts were burst in 40 mL of 5 mM MgCl_2_ and the spheroplast membranes (IM) were recovered by centrifugation at 15,000 rpm for 20 min.

Two membrane markers, the KDO (2-ketodeoxyoctonate) content for the OM and the NADH oxidase activity for the IM (de Maagd and Lugtenberg, 1986) were used to check the purity of each membrane fraction. The KDO content was 4.2/100 μg protein and 0.6/100 μg protein for the OM and IM, respectively. NADH oxidase activity was 760 and 17.6 U/mg protein/min for the IM and OM, respectively. The content of these markers demonstrates efficient separation and purity of the isolated OM and IM fractions.

### Determination of Membrane Fluidity

Membrane fluidity was determined by measuring the fluorescence polarization (*P*) of the DPH or TMA-DPH probe inserted into the membrane. *P* quantifies the degree of depolarization of light emitted by the embedded fluorescent probe and is an indirect measure of the membrane organizational state (Kuhry et al., 1985). *P* and membrane fluidity are inversely correlated. As the fluidity of the bacterial membrane decreases, *P* increases and vice versa (Lakowicz, 1999).

For all samples, fluorescence intensity (FI) measurements were performed using a FluoroMax^®^-Spex 4 Jovin Yvon (Horiba, NJ, United States) spectrofluorometer equipped with excitation and emission polarizers. The widths of the excitation and emission beam slits were set at 5 nm. FI were measured at excitation (λ_ex_) and emission (λ_em_) wavelengths for DPH and TMA-DPH set at 358 and 428 nm, respectively. The degree of polarization

*P* was calculated from the FI as follows (Equation 1):

$$P=\frac{I_{V\,V}-I_{V\,H}.G}{I_{V\,V}+I_{V\,H}.G}$$

where I_VV_ and I_VH_ are the FI of vertically and horizontally polarized light components emitted after excitation by vertically polarized light, respectively, and G = I_HV_/I_HH_ is the sensitivity factor of the detection system (Lakowicz, 1999).

*P*_TMA–DPH_ and *P*_DPH_ data were used to determine the short-term effects of cyclic temperature changes on the fluidity of *S. meliloti* membranes in WC. In turn, the *P*_DPH_ was used to determine the short-term effects of cyclic temperature changes on the fluidity of different fractions of *S. meliloti* membranes such as spheroplast (IM) and isolated OM. The long-term response (homeoviscous adaptation) was confirmed by the *P*_TMA–DPH_ and *P*_DPH_ measured in WC and multilamellar vesicles (MLV) prepared with lipids extracted from OM and IM membranes.

#### In Whole Living Cells

Following the procedures described by Trevors (2003), aliquots (25 mL) of *S. meliloti* cell suspension were collected at 28°C (control) and after each temperature change, harvested, washed in sterile 15 mmol L^–1^ Tris–HCl buffer (pH 7.0), and resuspended in the same buffer up to OD = 0.2 at 600 nm. For FI measurement, 1 μL of DPH (12 mmol L^–1^ stock solution in tetrahydrofuran) or TMA-DPH 12 mmol L^–1^ (stock solution in DMSO) were added to 3 ml of resuspended cells to obtain a final probe concentration of 4 μmol L^–1^ (a typical concentration used for microviscosity studies in model (Sanchez et al., 2007) and natural (England et al., 2003) membranes) and incubated for 10 min in the dark with magnetic stirring at 200 rpm.

#### In Spheroplast (Inner Membrane) and Outer Membrane

Three milliliters of a spheroplast solution (OD = 0.2 at 600 nm) obtained as described above from control cells (28°C) and from cells submitted to each temperature treatment were used to determine the FI of DPH with parallel and perpendicular oriented polarizers as already described for living cells. In addition, spheroplast from control cells were subjected to rapid changes in temperature (10 min), and the FI was determined to understand the rapid effects of temperature changes. The same tests were performed for OM samples with the difference that, for this, 3 mL of an OM suspension containing 0.5 mg/ml of protein were used.

#### In Multilamellar Vesicles of Lipids Extracted From Outer Membrane and Inner Membrane

Multilamellar vesicles were prepared according to Yamazaki et al. (1989). Briefly, 1 mL of Tris/HCl buffer (pH 7) was added to dry lipids extracted (see below) from OM or IM of *S. meliloti* control cell and cells subjected to each thermal treatment. The lipid suspension (3 × 10^–3^ M total lipids) was heated up to ∼40°C for 20 min and vortexed several times for approximately 5 min. Then, 1 μL of DPH was added to 3 mL aliquots to obtain a final probe concentration of 4 μmol L^–1^ and incubated for 10 min in the dark under stirring at 200 rpm.

### Incorporation of Labeled Acetate

A total of 0.5 μCi of sterilized 1-[^14^C]-acetate sodium salt (43 mCi mmol^–1^, New England Nuclear) was added to 25 mL bacterial cultures at the time of inoculation. Cells grown under these conditions were harvested by centrifugation at 8,000 *g* for 10 min in a Beckman Allegra 64R refrigerated centrifuge. Pellets were washed twice with 0.9 % w/v NaCl and used for subsequent studies.

### Total Lipid Extraction

Lipids were extracted from bacterial mass using a mixture of chloroform/methanol/water (2:1:0.2 v/v/v). The lower phase enriched in phospholipids was dried under a stream of N_2_ and dissolved in an appropriate volume of 2:1 (v/v) chloroform/methanol (Bligh and Dyer, 1959).

### Separation and Quantification of 1-[^14^C]-Labeled Phospholipids

Aliquots of OM and IM lipid extracts were analyzed by TLC using a 65:25:4 chloroform/methanol/water (v/v/v) solution as the running solvent. Lipids were detected with iodine vapors, and the separated bands were identified by comparing with authentic purified standards. Lipid bands were resolved properly (see Supplementary Figure 3).

The bands separated in the TLC plates were scraped, dispersed in 2 mL of scintillation cocktail (25%v/v Triton X-100, 0.3% w/v diphenyloxazole in toluene), and the radioactivity was measured using a liquid scintillation counter (Beckman LS 60001 C) at a 60% efficiency.

### Analysis of Fatty Acids by GC-MS

Fatty acid methyl esters (FAMEs) were obtained from *S. meliloti* OM and IM lipid extracts treated with 10% BF3 in methanol for 90 min at 100°C (Morrison and Smith, 1964).

FAMEs were analyzed by gas chromatography (GC) using an Agilent 7890B coupled to a Mass Spectrometer Agilent 5977A (MS) equipped with a ZB-WAX (30 mm × 0.25 mm ID) Zebron column. The following GC-MS conditions were used: injector temperature, 240°C; column temperature, 180°C, maintained for 30 min; increase of 5°C/min to 240°C, maintained for 10 min. Run time: 46 min. MS: full SCAN, 40–500. Injection volume: 1 μl. Split: 1:10.

FAMEs were identified by comparing the retention times of the peaks resolved in a run of the sample under study with those exhibited by the components of a mixture of commercial standards run in the same experimental conditions (Sigma Chemical Co., St. Louis, MO, United States) (Kates, 1972). Afterward, MS was used to confirm FA structures. Relative percentages of each FA were calculated.

### Assays of *S. meliloti–M. sativa* Interaction

*Sinorhizobium meliloti* strain L5-30/pDG71 (p*trp*-*Gfp*mut3) that constitutively produced green fluorescent protein (GFP) under the control of a *Salmonella enterica* serovar Typhimurium *trp* promoter (Gage, 2002) were assayed for their symbiotic phenotypes on *M. sativa*.

*M. sativa* cv. Monarca seeds were surface sterilized as described by Gage et al. (1996). The seeds were washed extensively with sterile water and allowed to germinate for 3 days in the dark. One seedling, 1–2 cm long, was placed in one plastic pot 5 cm in diameter and 10 cm high, filled with coarse-grain vermiculite sterilized in an autoclave twice for 2 h at 121°C. To test early interaction events between *S. meliloti* and alfalfa (radical hair curvature and infection thread formation) a culture of 200 mL of *S. meliloti* was grown to stationary phase at 28°C and then had applied the thermal treatment (10°C–40°C–10°C). After each step of the thermal cycle, an aliquot of 50 mL of culture was separated, centrifuged, and the biomass resuspended in physiological solution up to a 1 × 10^9^ CFU mL^–1^ density. The plants were inoculated with 1 mL of the bacterial suspension and were placed in a growth chamber (24°C with illumination for 16 h/day).

The plantlets were grown on N-free medium as described by Rolfe et al. (1980). After 7 and 14 postinoculation days (dpi), plants were removed and the roots visualized, at a 10 or 40 × magnification for nodules and infection thread, respectively, with a Nikon Eclipse 50*i* equipped with an epi-fluorescence attachment (Nikon Instruments Inc.).

### Statistical Analysis

All experiments were carried out in triplicate. One-way ANOVA was used to analyze the results. When ANOVA indicated a significant treatment effect, the least significant differences test (Tukey, *p* < 0.05) was applied to compare the mean values.

## Results

### Composition of Phospholipids and Fatty Acids of Outer Membrane and Inner Membrane Grown Under Control Conditions

**FIGURE 1:**
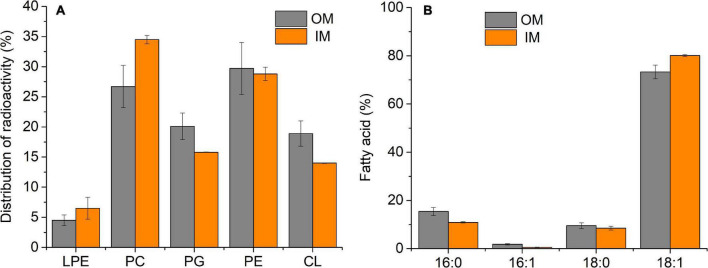
PL **(A)** and FA **(B)** composition of *S. meliloti* OM and IM at 28°C (control). 50 mL of *S. meliloti* culture with or without [^14^C]-acetate was grown to a stationary phase at 28°C. Afterward, OM and IM fractions were obtained. **(A)** Data from TLC of radioactively labeled PL bands scraped and quantified by liquid scintillation counting. **(B)** GC-MS analysis of FAME from total lipids. The percentage of each FA and PL is relative to the total defined as 100%. Values represent the mean ± sd of three independent experiments.

For both membranes under control conditions, the main PLs identified were PC, PE, phosphatidylglycerol (PG), and cardiolipin (CL) (Figure 1A); however, some differences could be observed between OM and IM. The highest percentage of PC (34.5%) was found in IM, whereas in OM, the main PL was PE (29.7%). The anionic PLs, PG and CL, were present at the highest percentages in OM (20.1 and 18.9%, respectively). Small amounts (<7%) of a PL ninhydrin positive, probably lysophosphatidylethanolamine (LPE), were detected in both OM and IM.

In the case of FA (Figure 1B), under control conditions, the content of 16:0, 16:1Δ^9^, 18:0, and 18:1Δ^11^ were qualitatively the same in both membranes, but OM presented a greater proportion of 16:0. Consequently, the unsaturated/saturated (U/S) FA ratio in OM was lower (U/S_OM_ = 3) than that of IM (U/S_IM_ = 4.1).

Although the 19:0 cycle FA is reported by several authors in lipids of *S. meliloti* (Tighe et al., 2000; Basconcillo et al., 2009), in this work, it has not been detected, probably due to the technique used to obtain FAMEs.

### Cyclic Temperature Changes Modify the Biophysical State of the Membranes

Temperature is one of the most studied environmental factors in terms of its effect on the fluidity of the membrane in bacteria of different genus. However, there are no documented data on the effects of successive changes in temperature within the same bacterial population. DPH and TMA-DPH are extensively used to probe the membrane organization by monitoring their fluorescence polarization (*P)*. Because DPH is a hydrophobic substance and TMA-DPH a derivative that, at the assayed pH, bears a net charge in its TMA moiety, they localize and, thus, sense the molecular order and mobility within the core and the polar head group regions of bilayers, respectively.

**FIGURE 2:**
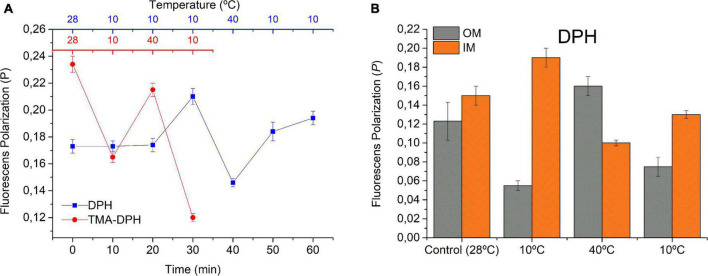
Effect of fast cyclic temperature changes on the fluorescence polarization. **(A)** Using DPH and TMA-DPH as fluorescent probes in whole *S. meliloti* cells and **(B)** using DPH in OM and IM. *S. meliloti* culture was grown to a stationary phase at 28°C, centrifuged, and the biomass resuspended in 15 mmol L^–1^ Tris–HCl buffer (pH 7.0) up to an OD600 nm = 0.2. The fluorescent membrane probes (DPH or TMA-DPH) 4 μmol L^–1^ were added to the resuspended culture (3 mL). Fluorescence polarization was measured in one aliquot at time = 0 min at 28°C (control sample) and after each successive temperature change. OM and IM (spheroplast) fractions from control cells were incubated with DPH probe 4 μmol L^–1^ and were subjected to rapid changes in temperature (10 min), and the fluorescence polarization was determined. Values represent the mean ± sd of three independent experiments.

Here, we evaluated the effect of rapid temperature changes on the *P_DPH_* and *P_TMA–DPH_* in WC of *S. meliloti* (Figure 2A) as well as in two subcellular fractions (IM and OM) (Figure 2B). WC exhibited a *P*_*DPH,28*_ = 0.17 in control conditions (28°C) and, after the initial incubation at 10°C for 30 min, the fluorescence polarization increased up to *P*_*DPH, 10i*_ = 0.21. This result can be interpreted as a greater rigidity in the membrane and lesser fluctuation of the acyl chains within the lipid bilayer. A subsequent temperature increase to 40°C led to a fast decrease in *P_DPH_*, reaching *P*_*DPH,40*_ = 0.14 after 10 min incubation, indicating an increase in the mobility of the probe within less ordered acyl chains and, thus, a higher membrane fluidity. Then, after 20 min incubation at 10°C, the final state in the cycle was reached. As expected, there was an increase in *P_DPH_* with respect to the previous state *(P_*DPH,10f*_* = 0.19) but slightly below the initial value of *P*_*DPH,10i*_.

The environment sensed by TMA-DPH in WC at the initial state showed a *P*_*TMA–DPH,28*_ ∼0.23, which was significantly higher than *P*_*DPH,28*_. Sequential 10 min incubation at 10°C, 40°C, and 10°C led *P*_TMA–DPH_ to change in the opposite direction with respect to the changes observed with DPH. Hence, *P*_TMA–DPH_ decreased up to *P*_*TMA–DPH,10i*_ = 0.16, increased up to *P*_*TMA–DPH,40*_ = 0.21, and then decreased again to *P*_*TMA–DPH,10f*_ = 0.12. Moreover, TMA-DPH responded faster than DPH to the initial cooling step.

*P*_DPH_ followed changes in the expected direction, increasing at low *T* and decreasing at high temperatures, suggesting that cyclic *T* changes (cooling–heating–cooling) induced a stiffening–fluidization–stiffening phenomenon in *S. meliloti*. However, the changes in *P*_TMA–DPH_ did not allow for a straightforward interpretation and required further considerations (see section “Discussion”). Additionally, because these experiments were performed in WC, the effects of DPH might have been reflecting the behavior of membranes as well as hydrophobic cell environments, such as lipid droplets as described previously for eukaryotic K562 leukemia cells (Balogh et al., 2011). Moreover, it is important to take into account that Gram-negative cells such as *S. meliloti* have a double membrane cell envelope. The dissection of the behavior of isolated OM and IM (Figure 2B) seemed to help in data interpretation. In control conditions, the values of *P*_DPH_ in OM and IM differed between one another with *P_*DPH, OM,28*_ < P_*DPH, IM,28*_*, and in general, they were lower than those found in WC. In control conditions, the *P*_DPH_,*_OM,28_* = 0.12. After 30 min exposure to 10°C, *P*_*DPH*_,*_OM_* decreased, resulting in *P*_*DPH*_,*_OM,10i_* = 0.05, and the opposite effect was observed in IM, where the *P*_*DPH*_,*_IM,28_* = 0.15 increased up to *P*_DPH_,*_OM,10i_* = 0.19. The difference *P*_*DPH*_,*_OM,10i_* < < *P*_DPH_,*_IM,10i_* widened if compared with the control at 28°C. The following step was the exposure to 40°C, which in OM, caused a considerable increase in OM (*P*_*DPH*_,*_OM,40_* = 0.16) and a decrease in spheroplasts (*P*_*DPH*_,*_IM,40_* = 0.10), leading to an inversion in the relationship between *P*_*DPH*_ of OM and IM, with *P_DPH_,_OM,40_* > *P*_*DPH, IM,40*_. Then, cooling up to 10°C again caused a decrease in OM (*P*_DPH_,*_OM,10f_* = 0.07) and, on the contrary, an increase in spheroplasts (*P*_*DPH*_,*_IM,10f_* = 0.13), leading to a new inversion of the relationship of *P*_DPH_ between both membranes. However, the difference *P*_DPH_,*_OM,10f_* < *P*_*DPH*_,*_IM,10f_* was less significant than *P*_DPH_,*_OM,10i_* < < *P*_*DPH*_,*_IM,10i_* and the absolute value of *P*_*DPH*_,*_IM,10f_* was lower than that of *P*_*DPH*_,*_IM,10i_*.

### Homeoviscous Adaptation of *S. meliloti* Membranes During Cyclic Temperature Changes

**FIGURE 3:**
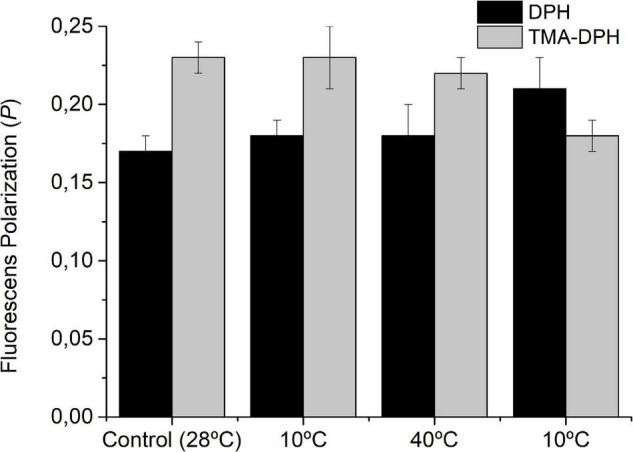
Effect cyclic temperature changes on the fluorescence polarization of DPH and TMA-DPH in WC after 24 h of thermal change. *S. meliloti* (100 mL) culture was grown to a stationary phase at 28°C and then the thermal changes applied (10°C–40°C–10°C) for 24 h incubation periods. After exposure to each temperature, one aliquot (25 mL) was centrifuged and the biomass resuspended in 15 mmol L^–1^ Tris–HCl buffer (pH 7.0) up to an OD600 nm = 0.2. The fluorescent membrane probe (DPH or TMA-DPH) 4 μmol L^–1^ was added to the resuspended culture (3 mL). Fluorescence polarization was measured at the same temperature from which the samples come. Values are the mean ± sd of three independent experiments.

To test the homeoviscous adaptation of *S. meliloti* to the effects of *T* changes on its fluidity, we measured the *P_DPH_* and *P_TMA–DPH_* in WC after 24 h incubation at each temperature (Figure 3). The bacterial suspension showed the same *P*_*DPH*_ and *P_TMA–DPH_* as the control after the initial incubation to 10 and 40°C, clearly indicating that *S. meliloti* has the adaptive mechanisms that allow its outer and cytoplasmic membrane to return to the viscosity level of the control after the *T*-induced perturbation. However, the exposure to 10°C for the second time, after being exposed to 40°C, impaired the return to the *P_DPH_* and *P_TMA–DPH_* values of the control. Thus, after the second exposure to 10°C, the *P_DPH_* values indicate that the IM remains more rigid compared with control cells (28°C) while *P_TMA–DPH_* indicates that the OM remains more fluid.

Because modifications in the lipid components are usually involved in the homeoviscous adaptation phenomenon, we studied the effect of *T* on the composition of PL and FA of the cellular envelope of *S. meliloti*.

### Effect of Cyclic Temperature Variations on the Composition of Phospholipid and FA From *S. meliloti* Outer Membrane and Inner Membrane

**FIGURE 4:**
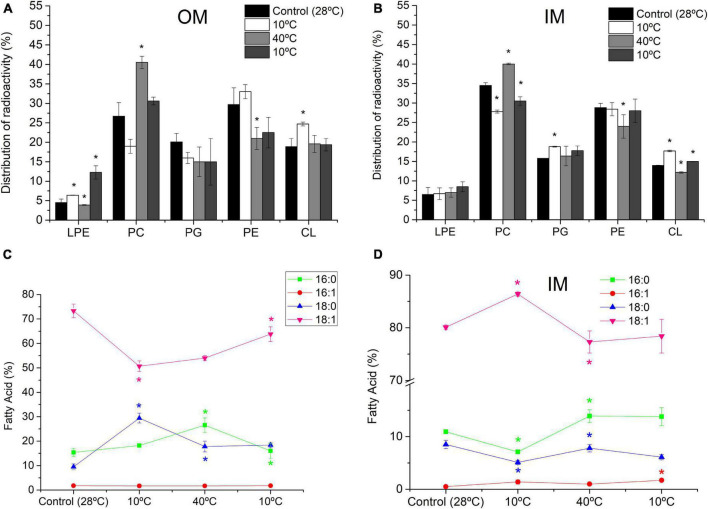
Effect of cyclic temperature variations on the composition of PL and FA from *S. meliloti* OM **(A,C)** and IM **(B,D)**. *S. meliloti* culture (200 mL) with or without [^14^C]-acetate was grown to a stationary phase at 28°C and then the thermal changes applied (10°C–40°C–10°C) every 24 h incubation period. After exposure to each temperature, a 50 mL aliquot of culture was separated and the biomass used to obtain OM and IM fractions. Panels **(A,B)** data from TLC of radioactively labeled PL bands scraped and quantified by liquid scintillation counting. Panels **(C,D)** GC-MS analysis FAME from total lipids. The percentage of each FA and PL is relative to the total defined as 100%. Values represent the mean ± sd of three independent experiments. Asterisks indicate statistically significant changes with respect to the previous stage.

Figure 4 depicts the effect of temperature changes on the composition of PL and FA of each *S. meliloti* membrane (further details are described in Supplementary Tables 1, 2). Thus, 10°C caused, with respect to the control (28°C), a decrease in PC (28.8%) and an increase in CL (30.7%) and LPE (42.2%) in OM (Figure 4A), whereas in IM, the decrease in PC (19%) was also accompanied by an increase in the anionic PLs PG (19%) and CL (26%) (Figure 4B). Also, the first exposure to 10°C in OM caused a decrease in 18:1 (30.7%) and an increase in the saturated 16:0 (18%) and 18:0 (209%) (Figure 4C). In the IM, the response was completely the opposite to that expected for a cooling process: The content of saturated 16:0 (34.8%) and 18:0 (40%) decreased, and 18:1 increased 7.8% (Figure 4D).

After the incubation at 40°C, both membranes exhibited an increase in PC levels (113% and 44% for OM and IM, respectively), whereas PE decreased (36% and 15% in OM and IM, respectively)—a completely opposite response to that observed for exposure at 10°C. In addition, we observed a strong decrease in CL (31%) in IM (Figure 4B). Changes in the FA levels were almost opposite between both membranes, increasing 16:0 (45%) and decreasing 18:0 (40%) in OM (Figure 4C) and increasing 16:0 and 18:0 (95.8% and 53%, respectively) and decreasing 18:1 (10%) in IM (Figure 4D).

When the same cell sample was exposed again for 24 h at 10°C, we observed a decrease in PC (24.4% and 23% for OM and IM, respectively) compared with the previous condition (Figures 4A,B). Moreover, a strong increase in LPE (215%) was observed in OM (Figure 4A). The FA response of OM in this condition was different from that found in the previous exposure at 10°C, increasing 18:1 (18%) and decreasing 16:0 (40%) with respect to the control (28°C). In IM, the exposure at 10°C for the second time increased up to 40% the 16:1 content with respect to the cells subjected 24 h at 40°C.

The changes in FA composition can be synthesized in the U/S ratio. After successive 24 h incubation periods at 10°C, 40°C, and 10°C, in the OM, the U/S_OM_ ratio varied from 3.0 in the control to 1.1, 1.2, and 1.9, respectively. Interestingly, the transition to 40°C, although it did not affect the U/S_OM_, a decrease in the mean length of the acyl chains of saturated FA was observed, by increasing 16:0 and decreasing. Underlying the process of homeoviscous adaptation is the stress-triggered catalytic activity of membrane-bound enzymes (Solís-Oviedo et al., 2012) and/or membrane sensors related to signal transduction mechanisms (Geiger et al., 2021). Thus, the membrane remodeling in composition and organization may operate as an on/off switch on the controlling mechanisms 18:0. In general, after the first 10°C incubation, the U/S_OM_ values are not in the line of compensating *T*-induced changes in membrane organization.

In turn, in IM, the U/S_IM_ of the control (4.1) changed in the following sequence, 7.2, 3.6, and 4.0 after successive 24 h incubation at 10°C, 40°C, and 10°C, respectively, along the thermal cycle. The variations in U/S_IM_ occurred in the direction expected to compensate for the *T*- induced changes in membrane microviscosity.

### Fluorescence Polarization of DPH in Outer Membrane, Inner Membrane, and Multilamellar Vesicles Prepared With Lipids Extracted From Samples Submitted to the Temperature Cycle

**FIGURE 5:**
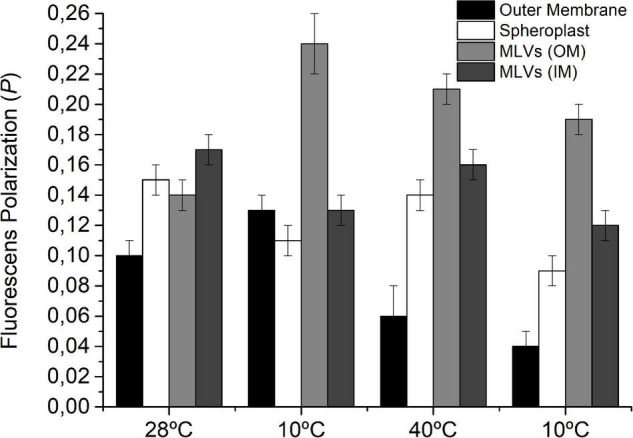
Fluorescence polarization of DPH in IM (spheroplast) and OM as well as in MLVs of lipids extracted from *S. meliloti* OM and IM after each temperature change. *S. meliloti* culture (200 mL) was grown to a stationary phase at 28°C and then the thermal changes applied (10°C–40°C–10°C) for 24 h incubation periods. After exposure to each temperature, one aliquot (50 mL) was separated, and OM and IM fractions were obtained. MLVs were prepared from lipids obtained of OM and IM. The fluorescent membrane probe (DPH) 4 μmol L^–1^ was added to the membranes and MLVs suspension (3 mL) and incubated to facilitate incorporation of the probe. Fluorescence polarization was measured at 25°C. Values are the mean ± sd of three independent experiments.

We measured the value of *P_DPH_* in isolated OM and spheroplasts (IM) as well as in MLVs prepared with the lipids extracted from OM and IM of cells previously subjected to successive 24 h incubation periods at 10, 40, and 10°C (Figure 5). The measurement of *P* in these systems after 24 h of exposure to each *T* allowed us to investigate if the modifications in the lipid composition were in line with restoring fluidity to optimal values after the initial disturbance. Besides this, MLVs are a more simplified system in which only the behavior of the lipid fraction (changes in *U/S* ratio) can be evaluated.

In the case of isolated OM, the *P_OM_* values were 0.10, 0.13, 0.06, and 0.04, respectively, for the control, 10°C, 40°C, and 10°C, whereas *MLV*_OM_ exhibited the following *P*_*DPH,MLV,OM*_ values for the control, 10°C, 40°C, and 10°C: 0.14, 0.24, 0.21, and 0.19, respectively. Using both systems, it can be appreciated that the OM presents a response ordering–disordering–ordering to the cooling–heating–cooling cycle, which might only be explained by the variations in *U/S_OM_* during the first exposure to 10°C with respect to the previous stage.

However, in IM, the *P*_IM_ values were 0.15, 0.11, 0.14, and 0.09, respectively, for the control, 10°C, 40°C, and 10°C, whereas in *MLV*_IM_, *P*_*DPH,MLV,IM*_ values were 0.17, 0.13, 0.16, and 0.12, exhibiting a response disordering–ordering–disordering that accompanies the observed variation in *U/S_IM_*.

### Implications of Changes Caused by Temperature on the Ability of *S. meliloti* to Interact With *M. sativa*

**FIGURE 6:**
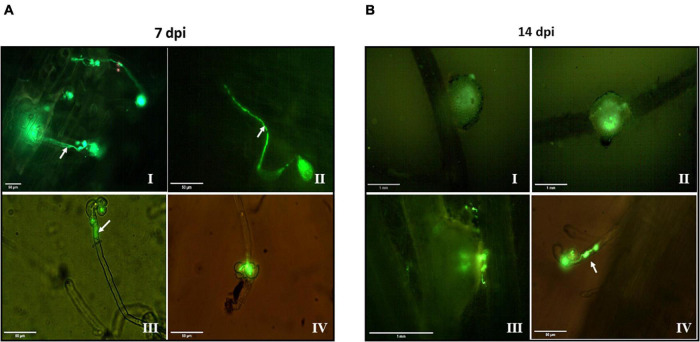
Effect of temperature changes on the symbiotic behavior of *Gfp*-expressing *S. meliloti* with roots of *Medicago sativa*. Alfalfa roots of 7 **(A)** and 14 **(B)** dpi with *Gfp*-expressing *S. meliloti* grown under control conditions **(I)** and exposed to 10°C **(II)**, 40°C **(III)**, and 10°C **(IV)**. Arrows indicate infection threads generated by *S. meliloti* within root hairs. A culture of *Gfp*-expressing *S. meliloti* (200 mL) was grown to stationary phase at 28°C and then the thermal treatment applied (10°C–40°C–10°C). After exposure to each temperature for 24 h, an aliquot of 50 mL of culture was separated, centrifuged, and the biomass resuspended in physiological solution up to an CFU mL^–1^ of 1 × 10^9^. Then an aliquot of that suspension (1 mL per plant) was used for inoculation tests. After 7 and 14 dpi, plants were removed, and the roots visualized in a fluorescence microscope at a 10 or 40 × magnification for nodules and infection thread respectively. Bars, 50 μm for panels **(A I–IV, B IV)**, 1 mm for panels **(B I–III)**.

We used *S. meliloti Gfp*-expressing cells in the different stages of the cycle (10°C–40°C–10°C) to perform interaction tests with *M. sativa* and evaluated the development of early symbiotic structures, such as infection threads after 7 dpi (Figure 6A) and their ability to evolve toward the nodule after 14 dpi (Figure 6B).

*Medicago sativa* seedlings inoculated with *S. meliloti* grown under control conditions exhibited threads of infection approximately between 5 and 7 dpi (Figure 6A-I) initiated from curled root hairs and extended through the epidermal cells, whereas nodular structures were detected microscopically at 14 dpi (Figure 6B-I).

The exposure to 10°C (Figures 6A-II,B-II) did not modify the bacteria’s symbiotic behavior with respect to the control. However, the subsequent exposure at 40°C delayed the symbiotic events as shown by the incipient infection threads observed at 7 dpi (Figure 6A-III). Additionally, at 14 dpi (Figure 6B-III) the nodular structures were in a formation stage earlier with respect to the previous condition. When *S. meliloti* was exposed to 10°C for a second time, threads were evidenced only after 14 dpi without the formation of microscopically visible nodular structures (Figure 6B-IV), reflecting serious problems to establish the symbiotic interaction with *M. sativa* at least until then.

## Discussion

In the present study, we put a dynamical insight on the capability of *S. meliloti* to respond in an adaptive manner to the fluctuations in a physical environmental variable, such as the temperature, which is crucial for controlling its development, growth, and viability.

The results obtained in the present work indicate that both *S. meliloti* membranes can sense differentially the cyclic changes in *T*. All the systems studied (WC, OM, and IM) respond dynamically to the changes in temperature, exhibiting a hysteresis along the cooling–heating–cooling cycle. The variation in *P*_DPH_ in WC and IM followed the expected directions, increasing upon cooling (more ordered, more anisotropic system) and decreasing upon heating (less ordered, more isotropic system). However, *P*_TMA–DPH_ in WC and those of *P*_DPH_ in isolated OM implied responses to *T* changes that occurred in the direction opposite to that expected. These results can be understood if we consider that OM, present in WC as well as in the isolated OM fraction, includes LPS in its outer leaflet. The polysaccharide moiety of LPS forms a hydrophilic layer close to the OM surface, which would represent a barrier that impairs the penetration of hydrophobic substances, such as DPH to the inner cell compartments and retain and concentrate hydrophilic substances such as TMA-DPH. This would explain the relatively low *FI*_DPH_ and high *FI*_TMA–DPH_ measured in O- containing samples (see Supplementary Figure 1). However, because the impairment would not be absolute in WC and is absent in IM, the DPH molecules that reach the IM would find an environment capable of exhibiting *P*_DPH_ changes that accompany the *T*-induced anisotropy dynamics (Figures 2A,B). Confirming this hypothesis, this behavior was not observed in OM but appeared in MLVs prepared with the lipids extracted from OM (isolated from control cells of *S. meliloti*) which lack the LPS (Supplementary Figure 2). The retention of TMA-DPH within the LPS layer implies that the *P*_TMA–DPH_ would be coupled to the dynamics of the polysaccharide moiety of LPS in the outermost part of the cell envelope. This hydrophilic environment can be envisioned as a macromolecular crowded media highly hydrated in which most of the water molecules are tightly bound (structured) to the hydrophilic surfaces (Clop et al., 2014). In these conditions, the properties of water as a solvent are reduced compared with bulk water and can be modulated by the physicochemical properties of the system (*T*, ionic strength, etc.). The solubility and, thus, the partitioning of TMA-DPH within this membrane region would be reduced at low temperature. We can envision that lowering *T*, TMA-DPH could be expelled from the membrane toward the isotropic bulk water, which is reflected by a lowering of *P*_TMA–DPH_, the opposite to what would be expected if this probe was located in a lipid bilayer. This rationale and the faster dynamics of a polymer compared with a lipid bilayer (Clop et al., 2014) also explains that the *T-*induced changes in *P*_TMA–DPH_ were faster than those of *P*_DPH_ (Figure 2A). Other photophysical phenomena should be considered to interpret the opposite behavior of *P*_DPH_ in OM and IM and that of *P*_TMA–DPH_. In this sense, the effect of decreasing the polarity and water availability of the environment (e.g., in the polymeric LPS regions at low temperature) can increase the emission lifetimes (|) of both probes, and in the case of TM-DPH, the transition angle (⟨) between the excitation and emission dipoles may also increase. According to the Perrin equation, both phenomena contribute to the *P* decrease within the LPS region of OM when *T* becomes lower (see Supplementary Material for details).

It is vitally important that bacteria maintain the fluidity of their membranes at optimal values to ensure physiological homeostasis and the integrity of all the processes that occur in them. This fluidity control process, called homeoviscous adaptation, was first demonstrated in *E. coli* by observing that membrane fluidity remains relatively constant at various temperatures (Sinensky, 1974). In Gram-negative WCs, it is very difficult to test the homeoviscous adaptation of each membrane due to the lack of knowledge of the location of the fluorescent probes that are used to measure fluorescence polarization. In this work, we were able to verify that DPH is a good indicator of IM behavior. Our results also highlight that, for the correct interpretation of data obtained with DPH in OM and with TMA-DPH in both OM and IM (atypical at first sight), it is required to consider the physicochemical properties of the environment in which they are located, their partitioning properties, and the photophysical behavior of these probes (see Supplementary Material). Thanks to these results, we differentially monitor the behavior of each *S. meliloti* membrane during the thermal cycle in WC. After 24 h of exposure to the first cooling and subsequent heating, both membranes presented *P*_DPH/TMA–DPH_ values similar to the control (Figure 3), indicating that, under these conditions, there are compensatory mechanisms that allow achieving homeoviscous adaptation. At the end of the thermal cycle, the bacteria seemed to have weakened the control on its membrane fluidity.

The fluidity control was accompanied by modifications in the composition of PL; thus, the content of PC was strongly regulated at all temperatures tested in both OM and IM. Note that PC can be synthesized from PE *via* successive methylations catalyzed by pmt enzymes (Sohlenkamp and Geiger, 2016). This may explain that, in both OM and IM, mainly the exposure to 40°C led to the increase in PC accompanied by a decrease in PE. The role of PLs, mainly PC, in relation to different environmental conditions in *Bradyrhizobium* TAL1000 and SEMIA6144 (Paulucci et al., 2011, 2013; Cesari et al., 2018) and in *S. meliloti* (present work) may be related to the stabilization of the membrane because of the important function of phospholipids such as PC to form a stable lamellar structure of cell membranes, facilitated by its geometrical shape and physicochemical properties (Boumann et al., 2006). We propose that, in these types of bacteria, PC variations may be a universal response. This is not the case of the model bacterium *E. coli*, which lacks PC in the membrane (Geiger et al., 2013).

In general terms, the *T* changes applied to *S. meliloti* cause the same modifications in the composition of PL in OM and IM. An exception was the increase in the thermal-induced content of LPE in OM during the second exposure to 10°C, which was a distinctive feature between both membranes. LPE derives from the PLA-catalyzed hydrolysis of PE, and this enzyme is present in the OM of Gram-negative bacteria (Song and Rhee, 2001; Beney et al., 2007). The role of lysophospholipids in bacteria remains poorly studied; it has been proposed that the inverted cone shape of this lipid molecule could alleviate the curvature stress caused on the membrane in certain situations (Zheng et al., 2017). LPE was also found to become dominant in the OM of *Y. pseudotuberculosis* and *B.* SEMIA6144 after shifting the growth temperature from 8 to 37°C and under water deficit, respectively (Davydova et al., 2016; Cesari et al., 2018). Although both exposures to 10°C caused increased content of LPE in OM, the effect was more significant in the second exposure at 10°C (after the incubation at 40°C). Whereas in the first incubation at 10°C, the Δ*T* was −18°C compared with the previous state, the wider Δ*T* (−30°C) of the second exposure to 10°C may be responsible for the greater disturbance observed in the OM.

The control of the degree of unsaturation of FA is one of the mechanisms widely used by cells to maintain lipid packing, membrane microviscosity, and water permeability (Lande et al., 1995; de Mendoza and Pilon, 2019). In *S. meliloti*, the variations in *U/S_IM_* occurred in the direction expected to compensate for the *T*-induced changes in membrane microviscosity because a decrease in *U/S* is expected to result in stabilization of membranes after heating. Surprisingly, after 24 h at 40°C, *U/S_OM_* did not show variations with respect to initial 10°C, indicating the presence of other mechanisms to compensate for the perturbation induced by *T* in OM, such as the shortening of the acyl chain due to a decrease of 18:0 from 16:0. It is important to note that the change in the value of *U/S_OM_* during the transition from 40 to 10°C occurs precisely in the opposite direction to that which would be expected to counteract the fluidizing effect; this could explain why, in this condition, *S. meliloti* is unable to restore the microviscosity of OM. From the few bibliographic data available on the response of OM from Gram-negative bacteria to temperature changes, the work of Davydova et al. (2016) can be cited, and they show a difference between OM and IM of *Y. pseudotuberculosis* subject to a temperature shock from 8 to 45°C. They found that IM was the membrane fraction that exhibited some features of thermal adaptation (*U/S* ratio decreased from 2.3 to 1.5), whereas in OM, although the authors claim that there were no significant changes in the composition of FA, the response observed was clearly contrary to that of IM. It is important to highlight that the biochemical changes observed are due to thermal cycling and not to bacteria aging because no significant variations were observed in FA and PL composition of *S. meliloti* under control conditions (28°C) when it was analyzed at different times (see Supplementary Table 3).

The *P* data obtained in MLVs prepared with the lipids extracted from OM and IM allow establishing a direct correlation between the lipid composition and the response to the *T* disturbance in the membrane. In conjunction, variations in FA and PL compositions indicate that OM and IM have their own mechanisms to respond to thermal perturbation and maintain their fluidity at optimal values, in IM the control of the degree of unsaturation being the most important. The vision of *S*. *meliloti* from this perspective allowed us to highlight the differences in the response to *T* of WC and the isolated OM and IM. Furthermore, considering other Gram-negative bacteria species in the response to *T* (Paulucci et al., 2011, 2013, 2015), it can be suggested that IM is the most appropriate target to understand adaptations to thermal-induced perturbations. It is also worth noting that, although during the second exposure at 10°C, the fluidity of the *S. meliloti* membranes was not restored to control values, IM responded adequately. However, it seems that these changes did not allow reaching an optimum fluidity, probably because the long-time incubation at 40°C affected the cell viability and plasticity of their membranes to respond.

Underlying the process of homeoviscous adaptation is the stress-triggered catalytic activity of membrane bound enzymes (Solís-Oviedo et al., 2012) and/or membrane sensors related to signal transduction mechanisms (Geiger et al., 2021). Thus, the membrane remodeling in composition and organization may operate as an on/off switch on the controlling mechanisms.

The *S. meliloti* inability to control the fluidity of both membranes in the second exposure to 10°C was correlated with a delay in the formation of early symbiotic structures, such as infection threads and nodules, with respect to the other conditions, demonstrating that that maintenance of envelope integrity in *S. meliloti* is critical to achieve an effective symbiosis. The cell envelope of rhizobia is considered an important player during the establishment of symbiosis during the initial exchange of signals between the bacteria and the root (Hubac et al., 1994) as well as during their survival within the infection thread (Scheidle et al., 2005) and then in the symbiosome (Bolaños et al., 2004). Despite this and even though some components of the bacterial surface are described as essential in these processes (polysaccharides, LPS), the maintenance of integrity of the membranes after *T* disturbance had not yet been investigated in relation to the process of symbiosis.

Considering the limited available information in rhizobia on the OM and IM composition, its role in adaptation to stressful conditions, and in the symbiotic process, the data presented here could be a starting point for the design of bacteria with permanent changes in lipid composition through cell membrane engineering methods.

## Data Availability Statement

The raw data supporting the conclusions of this article will be made available by the authors, without undue reservation.

## Author Contributions

NP, MD, and MP designed the research and coordinated the study. NP carried out the experiments. MB and AC collaborated with fluorescence anisotropy measurements and the interpretation of the data. NP and MP wrote the manuscript. All authors contributed to the article and approved the submitted version.

## Conflict of Interest

The authors declare that the research was conducted in the absence of any commercial or financial relationships that could be construed as a potential conflict of interest.

## Publisher’s Note

All claims expressed in this article are solely those of the authors and do not necessarily represent those of their affiliated organizations, or those of the publisher, the editors and the reviewers. Any product that may be evaluated in this article, or claim that may be made by its manufacturer, is not guaranteed or endorsed by the publisher.
