# Supplementary material for: Membrane Homeoviscous Adaptation in Sinorhizobium Submitted to a Stressful Thermal Cycle Contributes to the Maintenance of the Symbiotic Plant–Bacteria Interaction
Source: Front Microbiol. 2021 Dec 17;12:652477. doi: 10.3389/fmicb.2021.652477 (PMC8718912; doi:10.3389/fmicb.2021.652477)
Supplement: Supplementary file 1 [file Data_Sheet_1.pdf]

# *Supplementary Material*

## MEMBRANES HOMEOVISCOUS ADAPTATION IN *SINORHIZOBIUM* SUBMITTED TO A STRESSFUL THERMAL CYCLE CONTRIBUTES TO THE MAINTENANCE OF THE SYMBIOTIC PLANT-BACTERIA INTERACTION.

Natalia Soledad Paulucci[1,2*], Adriana Belén Cesari[1,2], María Alicia Biasutti[3,4], Marta Susana Dardanelli[1,2*], María Angélica Perillo[5,6*]

[1] Facultad de Ciencias Exactas, Físico-Químicas y Naturales, Departamento de Biología Molecular, Universidad Nacional de Río Cuarto, Río Cuarto, Argentina

[2] Instituto de Biotecnología Ambiental y Salud (INBIAS), CONICET, Río Cuarto, Argentina

[3] Facultad de Ciencias Exactas, Físico-Químicas y Naturales, Departamento de Química, Universidad Nacional de Río Cuarto, Río Cuarto, Argentina

[4] Instituto para el Desarrollo Agroindustrial y de la Salud (IDAS), CONICET, Río Cuarto, Argentina

[5] Facultad de Ciencias Exactas, Físicas y Naturales, ICTA, Departamento de Química, Cátedra de Química Biológica, Universidad Nacional de Córdoba, Córdoba, Argentina,

[6] instituto de Investigaciones Biológicas y Tecnológicas (IIBYT), CONICET, Córdoba, Argentina

***Corresponding Authors**
npaulucci@exa.unrc.edu.ar
mdardanelli@exa.unrc.edu.ar
mperillo@unc.edu.ar

**CONTENTS** (S1 to S8 refer to page numbers)

S1 - Title, authors, affiliations and description of supplementary material content.

S2 - Photophysics of DPH and TMA-DPH.

S3 - Fluorescence intensity of DPH and TMA-DPH in outer and inner membrane of *S. meliloti*.

S4 - Effect of cyclic temperature changes in the fluorescence polarization of DPH in multilamellar vesicles from outer membrane lipids of *S. meliloti*.

S5 - Thin layer chromatography stained with $I_2$ vapor

S6 - Effect of cyclic temperature changes (10ºC-40ºC-10ºC) on the phospholipid composition of *S. meliloti* 1021 outer and inner membranes.

S7 - Effect of cyclical temperature changes (10ºC-40ºC-10ºC) on the fatty acid composition of *S. meliloti* 1021outer and inner membranes.

S8 –Effect of bacteria aging on the fatty acid (a) and phospholipid (b) composition of *S. meliloti* under control conditions (28ºC).

## PHOTOPHYSICS OF DPH AND TMA-DPH

Theoretically, $P$ can vary between -0.33 ≤$P_0$≤ 0.5 according to Eq. 1

$$P_0 = \frac{3.\cos(\alpha)-1}{\cos(\alpha)+3} \qquad (1)$$

Being DPH a linear molecule with parallel relative orientations of the excitation and emission transition dipoles, corresponding to an angle between both dipoles $\alpha$=0°, it can exhibit a maximal fluorescence polarization value $P_{0,max,DPH}$ = 0.5. In the case of TMA-DPH, the $P_{0,max,TMA\text{-}DPH}$=0.469 is due to the non-parallel excitation and emission transition dipoles ($\alpha \cong$ 18°) (Muller et al, 1996)

Measured $P$ values in real systems is defined by the Perrin equation (Eq.2):

$$P = \frac{P_0}{1+\frac{\tau}{\tau_0}} \qquad (2)$$

This shows that $P$ depends on the relationship between the lifetime of fluorescence emission ($\tau$, usually within the ns scale) as well as on the scrambling of orientations due to the tumbling of the whole fluorescent molecule or to the rotation of only its fluorescent part (defined by the rotational correlation time $\tau_0$). So, the rigidity or fluidity of the environment will impair (high $P$) or allow (low $P$), respectively, the tumbling or rotation of the fluorescent probe.

Other aspects to take into account when interpreting $P$ values are the effects of media polarity. On the one hand, the angle $\alpha$ between the absorption and emission transition dipole moments can decrease in polar solvents by more than a factor of two thus increasing $P_0$ (Belfield et al., 2007). On the other hand, water molecules act as a dynamic quencher of the excited states of DPH. In a low polarity environment with low water content, it was observed a constancy of the absorption and a decrease in the intensity and in the lifetime of the excited state of DPH. At high water content both absorption and emission are significantly low (Graceto et al., 2010). So, within a certain range of low hydration levels it can be expected to decrease $P$ values in decreasing water availability conditions.

Muller, J.M., Harryvan, D.H., Verhagen, J.C.D., van Ginkel, G., and van Faassen, E.E. (1996). The orientation of the transition dipole moments of TMA-DPH. embedded in a poly (vinyl alcohol) film. Chemical Physics, 211, 413-420.

Belfield, K.D., Bondar, M.V., Kachkovsky, O.D., Przhonska, O.V., and Yao, S. (2007). Solvent effect on the steady-state fluorescence anisotropy of two-photon absorbing fluorene derivatives. Journal of Luminescence, 126, 14–20.

Gracetto, A.C., Batistela, V.R., Caetano, W., de Oliveira, H.P.M., Santos,W.G., Cavalheiro, C. C.S., and Hioka, N.(2010). Unusual 1,6-Diphenyl-1,3,5-hexatriene (DPH) Spectrophotometric Behavior in Water/Ethanol and Water/DMSO Mixtures. J. Braz. Chem. Soc, 21, 1497-1502.

**Figure S1. Fluorescence intensity of DPH and TMA-DPH in outer and inner membrane of**

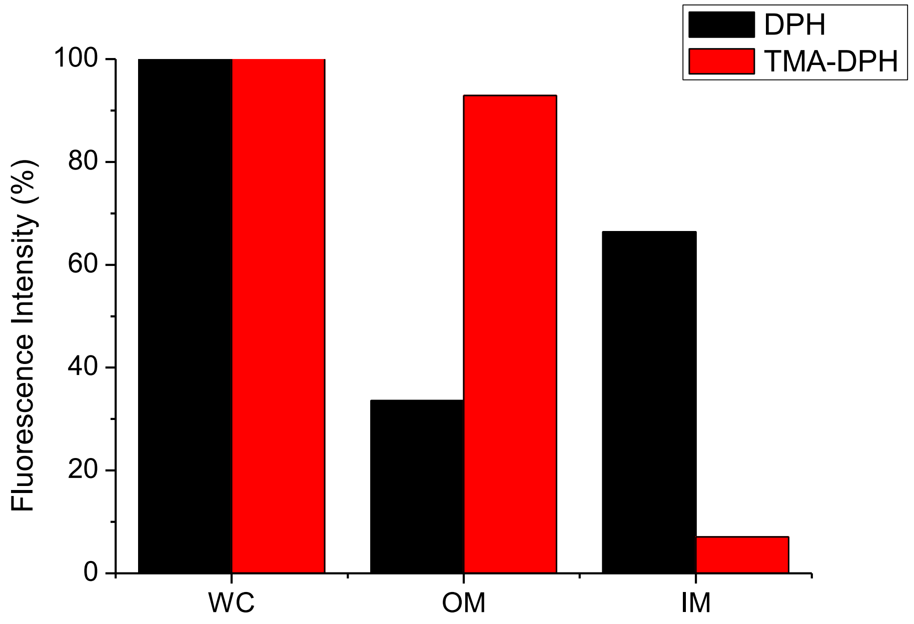


***S. meliloti*** **relative to the intensity measured in whole cells (100%).**

*S. meliloti* culture (100 mL), was grown to a stationary phase at 28ºC. After it was centrifuged and the biomass resuspended in physiological solution up to an OD620nm = 1. The fluorescent membrane probes (DPH or TMA-DPH) 25 µmol.$L^{-1}$ was added to the resuspended culture (50mL) and incubated in darkness to facilitate incorporation of the probe (20 min at 28ºC). Then, the cultures were centrifuged and the biomass used to obtain outer and inner membrane fractions. Then an aliquot of each membrane's suspension (3 mL) was used to measure fluorescence intensity. The graph is representative of three independent experiments.

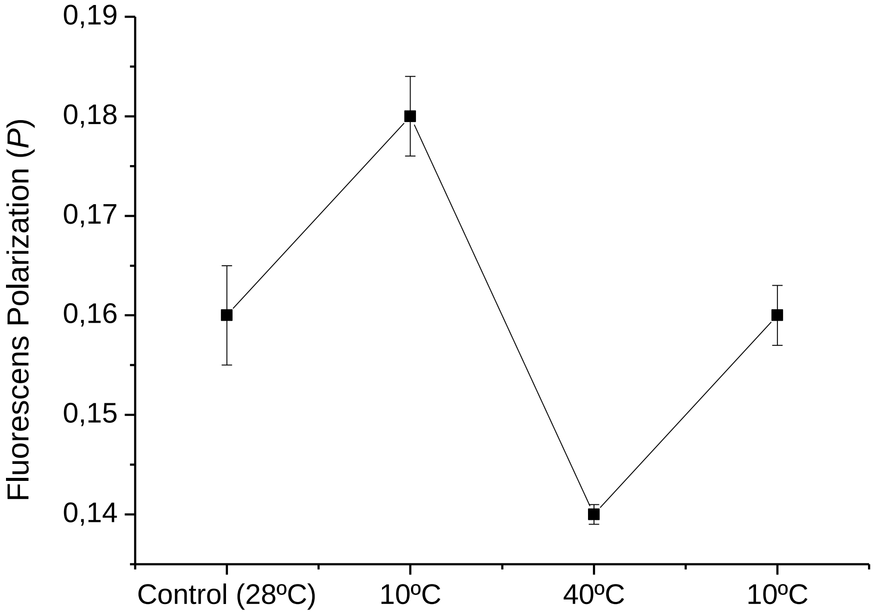


**Figure S2. Effect of cyclic temperature changes in the fluorescence polarization of DPH in multilamellar vesicles prepared from outer membrane lipids of *S. meliloti.***

Multilamellar vesicles were prepared from lipids obtained of OM control cells and incubated with DPH probe 4 µmol.$L^{-1}$ for 10 min in darkness. Then, were subjected to rapid changes in temperature (10 min) and the fluorescence polarization was determined. Values represent the mean of three independent experiments.

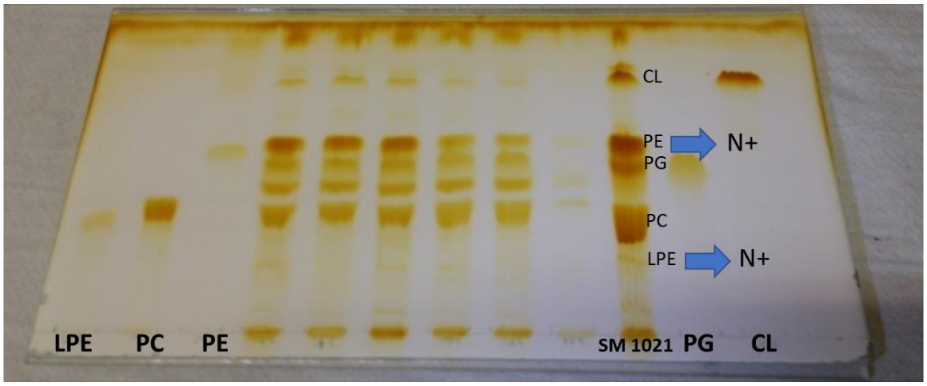


**Figure S3. Thin layer chromatography stained with $I_2$ vapor.** From left to right, LPE, PC, PE, PG and CL (standards); SM 1021, total phospholipids of *S. meliloti* 1021separated by TLC following the methodology indicated in the M&M section; N+, positive Ninhydrin bands. Samples run in lines 4-8 do not correspond to *S. meliloti*.

**Table S1: Effect of cyclic temperature changes (10ºC-40ºC-10ºC) on the phospholipid composition of *S. meliloti* 1021 outer and inner membranes.**

| Subcellular fraction | Phospholipid | Temperature (ºC) 28 | 10 | | 40 | | 10 | |
|---|---|---|---|---|---|---|---|---|
| | | Composition (%) | Composition (%) | Change (%) | Composition (%) | Change (%) | Composition (%) | Change (%) |
| ***Outer Membrane*** | **CL** | 18.9±2.1 | 24.7±0.5* | + 30.7 | 19.6±2.2 | | 19.4±1.6 | |
| | **PE** | 29.7±4.3 | 33.0±1.8 | | 21.0±2.8 | – 36 | 22.5±3.9 | |
| | **PG** | 20.1±2.2 | 16.9±1.4 | | 15.0±3.8 | | 15.0±6 | |
| | **PC** | 26.7±3.5 | 19.0±1.8 | – 28.8 | 40.5±1.6 * | + 113 | 30.6±1.0 | – 24.4 |
| | **LPE** | 4.50±0.9 | 6.40±0.07* | + 42.2 | 3.90± 0.1* | – 39 | 12.3±1.7 * | + 215 |
| ***Inner Membrane*** | **CL** | 14.0±0.05 | 17.7±1.7* | + 26 | 12.2±0.2* | – 31 | 15.0±0.05* | + 23 |
| | **PE** | 28.8±1.1 | 28.4±1.7 | | 24.0±3.0 | – 15 | 28.0±3.0 | |
| | **PG** | 15.8±0.05 | 18.8±0.1* | + 19 | 16.4±2.5 | | 17.8±1.2 | |
| | **PC** | 34.5±0.7 | 27.8±0.4* | – 19 | 40.0±0.2* | + 44 | 30.5±1.1* | – 23 |
| | **LPE** | 6.50±1.8 | 6.70±1.5 | | 7.00±1.2 | | 8.50±2.0 | |

A culture of 200 mL of *S. meliloti* with the aggregate of $C^{14}$ acetate, was grown to stationary phase at 28ºC and then applied the thermal treatment (10ºC-40ºC-10ºC). After exposure to each temperature for 24 h,50 mL of culture was separated, centrifuged and the biomass used to obtain outer and inner membrane fraction. The radioactively labeled samples were used for the analysis of the PL by TLC and subsequent quantification of the radioactivity of each fraction in a liquid scintillation counter. The percentage of each PL is relative to the total PL defined as 100%.
Values represent the mean ± SEM of three independent experiments. * Indicates a statistically significant difference with respect to the previous condition ($p$<0.05).

**Table S2: Effect of cyclical temperature changes (10ºC-40ºC-10ºC) on the fatty acid composition of *S. meliloti* 1021outer and inner membranes.**

| **Subcellular Fraction** | **Fatty Acid** | **Temperature (ºC)** | | | | | | |
|---|---|---|---|---|---|---|---|---|
| | | **28** | **10** | | **40** | | **10** | |
| | | **Composition (%)** | **Composition (%)** | **Change (%)** | **Composition (%)** | **Change (%)** | **Composition (%)** | **Change (%)** |
| ***Outer Membrane*** | **16:0** | 15.4±1.6 | 18.2±0.8 | | 26.5±3.0* | + 45 | 16.0±3.0* | – 40 |
| | **16:1$\Delta^{9}$** | 1.80±0.3 | 1.70±0.7 | | 1.70±0.5 | | 1.80±0.3 | |
| | **18:0** | 9.50±1.2 | 29.4±2.0* | + 209 | 17.8±2.2* | – 40 | 18.4±0.9 | |
| | **18:1 $\Delta^{11}$** | 73.3±2.8 | 50.7±2.2* | – 30.7 | 54.0±1.0 | | 63.8±3.0* | + 18 |
| | ***U/S* [a]** | **3.0** | **1.1** | | **1.2** | | **1.9** | |
| ***Inner Membrane*** | **16:0** | 10.9±0.3 | 7.10±0.8* | – 34.8 | 13.9±1.2* | + 95.8 | 13.8±1.7 | |
| | **16:1$\Delta^{9}$** | 0.50±0.1 | 1.40±0.4 | | 1.00±0.1 | | 1.70±0.1* | + 41 |
| | **18:0** | 8.50±0.8 | 5.10±0.4* | – 40 | 7.80±0.7* | + 53 | 6.10±0.5 | |
| | **18:1 $\Delta^{11}$** | 80.1±0.4 | 86.4±0.4* | + 7.8 | 77.3±2.1* | – 10 | 78.4±3.2 | |
| | ***U/S* [a]** | **4.1** | **7.2** | | **3.6** | | **4.0** | |

A culture of 200 mL of *S. meliloti* was grown to stationary phase at 28ºC and then applied the thermal treatment (10ºC-40ºC-10ºC). After exposure 24 h to each temperature,50 mL of culture was separated, centrifuged and the biomass used to obtain outer and inner membrane fraction. FAME were obtained from total lipids and analyzed by GC-MS. The percentage of each FA is relative to the total FA defined as 100%. Values represent the mean ± SEM of three independent experiments.
a Ratio between the sums of unsaturated and saturated FA (U/S).
* Indicates a statistically significant difference with respect to the previous condition ($p$<0.05)

**Tables S3. Effect of bacteria aging on the fatty acid (a) and phospholipid (b) composition of *S. meliloti* under control conditions (28ºC).**

(a)

| FA | FA (%) mean +/- sd (n=2) | |
|---|---|---|
| | 0 h | 72 h |
| 16:0 | 6.5±0.9 | 8.6±0.8 |
| 16:1Δ[9] | 1.2±0.3 | 1.1±0.2 |
| 18:0 | 8.6±0.8 | 10.3±0.9 |
| 18:1Δ[11] | 83.6±6.5 | 80.0±5.0 |

(b)

| PL | PL (%) mean +/- sd (n=2) | |
|---|---|---|
| | 0 h | 72 h |
| CL | 11.3±1.4 | 13.6±0.9 |
| PG | 17.6±2.3 | 18.2±3.0 |
| PE | 33.6±4.0 | 31.1±3.3 |
| PC | 30.8±3.6 | 32.0±2.8 |
| LPE | 5.7±0.5 | 5.0±1.0 |

Samples were analyzed at different times and no significant variations were found in either FA or PL composition. This indicates that the control used in the present study allows us realize that the biochemical and biophysical changes observed are due to thermal cycling and not to bacteria aging.
FA, fatty acid; PL, phospholipid, CL, cardiolipin; PG, phosphatidylglycerol; PE, phosphatidylethanolamine; PC, phosphatidylcholine; LPE, lysophosphatidylethanolamine.
